# Aminomethylphosphonic Acid (AMPA), a Glyphosate Metabolite, Decreases Plasma Cholinesterase Activity in Rats

**DOI:** 10.3390/jox14020035

**Published:** 2024-05-07

**Authors:** Jesús Chávez-Reyes, Fernando Saráchaga-Terrazas, Oliver Alejandro Colis-Arenas, Carlos H. López-Lariz, Carlos M. Villalón, Bruno A. Marichal-Cancino

**Affiliations:** 1 Department of Physiology and Pharmacology, Centre of Basic Sciences, Autonomous University of Aguascalientes, Ciudad Universitaria 940, Aguascalientes 20100, Mexico; jesus.chavezr@edu.uaa.mx (J.C.-R.); carlos.lopez@edu.uaa.mx (C.H.L.-L.); 2 Department of Medicine, Centre of Health Sciences, Autonomous University of Aguascalientes, Ciudad Universitaria 940, Aguascalientes 20100, Mexico; 3 Department of Pharmacobiology, Centre for Research and Advanced Studies, the National Polytechnic Institute (Cinvestav-Coapa), Czda. de los Tenorios 235, Col. Granjas-Coapa, Deleg. Tlalpan, Mexico City 14330, Mexico; cvillalon@cinvestav.mx

**Keywords:** aminomethylphosphonic acid (AMPA), glyphosate, plasma, acetylcholinesterase activity, plasma cholinesterase

## Abstract

Glyphosate, a widely used herbicide, is linked to a plethora of deleterious effects in both clinical and preclinical studies. Nevertheless, the effects of its main metabolite, aminomethylphosphonic acid (AMPA), whose half-life in soil is even longer than that of glyphosate, have been little explored. On this basis, as a first approach, in this work, we report that intraperitoneal (i.p.) administration of AMPA or glyphosate (at 10, 56, and 100 mg/kg) decreased, to a similar extent, plasma cholinesterase (ChE) activity in acutely exposed rats. Moreover, we designed an experimental protocol to analyze and compare the effects of AMPA and glyphosate on human plasma ChE activity; this protocol consisted of adding these compounds to human plasma to subsequently test the effects of this plasma on the contraction to acetylcholine (ACh) in the frog rectus abdominis muscle (an indirect estimate of ChE activity). Accordingly, this muscular contraction to ACh was evaluated before and after pre-incubation of ACh with (i) plasma alone, (ii) plasma with AMPA, and (iii) plasma with glyphosate. Our results indicate that AMPA, like glyphosate, decreased ChE activity in the plasma of rats (when given i.p.) and humans (when added in vitro), suggesting that both xenobiotics may exert similar toxicological effects.

## 1. Introduction

Glyphosate-based herbicides (GBHs) are the most popular synthetic herbicides employed as weed controllers [1]. Indeed, once glyphosate resistance crops arrived, use of GBHs increased exponentially, making them the most successful herbicidal compounds in the world [2]. As mammals lack 5-enolpyruvylshikimate-3-phosphate synthase, the target enzyme of glyphosate in plants [3], and because of its hypothetic low absorption [4], these compounds were considered relatively safe. Nevertheless, several reports have suggested multiple toxicological effects in preclinical and clinical studies [5]. Remarkably, some of these studies have reported teratogenic effects in the most dramatic cases [6], but also discrete alterations in cognition and behaviour [7], probably by a negative interaction with the enzyme acetylcholinesterase (AChE) [8]. In this regard, it has been shown that subchronic and chronic exposure to glyphosate decreased the activity of AChE in brain samples of rodents [9,10].

Interestingly, when deposited in soil, glyphosate can be degraded mainly into aminomethylphosphonic acid (AMPA), mostly by microbial action, increasing its half-life up to 958 days, while that of glyphosate is only up to 280 days [11]. Moreover, AMPA has been detected in (i) the blood of patients after accidental ingestion of GBHs [12]; (ii) urine samples from Mexican farm workers [13] and Californian residents living near glyphosate-application zones, with the latter associated with liver and cardiometabolic disorders in childhood [14]; and (iii) brains of mice receiving pure glyphosate by the oral route [15]. With these lines of evidence and the idea that AMPA can be produced by humans or rodents via intestinal microbiomes [16], it is striking to note that most of the biological effects of AMPA remain essentially unknown. Moreover, as AMPA has a larger half-life in soils compared to glyphosate, its levels in roots, crops, and water sources should be measured in areas where GBHs are still used to estimate potential toxicological effects.

A very recent report suggests that AMPA may be produced from other industrial activities nonrelated to glyphosate, including photodegradation of the amino-polyphosphonates extensively used in households as detergents and also employed as complexing agents in textile and paper industries [17]. Given the widespread use and permanence of AMPA in the environment, it is mandatory to study its potential toxicological profile in exposed organisms, industrial sources, and potential chemical environmental interactions. Indeed, we have recently reported that rats acutely exposed to glyphosate and AMPA showed a similar decrease in the activity of both brain and muscular acetylcholinesterase [18]. On this basis, the present study was conducted to analyze and compare the effects of acute exposure to AMPA and glyphosate on plasma cholinesterase (ChE) activity under two experimental conditions, namely (i) in the plasma obtained from rats intraperitoneally (i.p.) administered with these compounds; and (ii) in human plasma where these compounds were added in vitro to subsequently test the effects of this plasma on the contraction to acetylcholine (ACh) in the frog rectus abdominis muscle (i.e., an indirect estimate of ChE activity in human plasma).

## 2. Material and Methods

### 2.1. Materials

Glyphosate analytical grade (N-1233-250 mg, purity 99.5%) was purchased from Chemservice (West Chester, PA, USA). AMPA (324817-1G, purity 99%), acetylcholine chloride (A2661-100G), and the AChE activity assay Kit (MAK119, whose substrates may be susceptible to the action of both butyrylcholinesterase and acetylcholinesterase) were purchased from Sigma-Aldrich (St. Louis, MA, USA). To avoid degradation, fresh solutions were prepared on demand using injectable water as a vehicle. The concentrations or doses mentioned in this article refer to their respective free base.

### 2.2. Animals and Protocol of Intoxication

Forty-nine healthy Sprague Dawley (*Rattus norvegicus*) female rats (6–8 weeks old, 220–240 g) obtained from the vivarium of the Autonomous University of Aguascalientes were randomly divided using a blind method into 7 groups (*n* = 7 rats per group). The rats were maintained at 22 ± 2 °C on a 12 h-light/dark cycle (lights on at 8:00 h), having access to standard rodent food (Purina^®^) and water ad libitum.

Each group of rats received an intraperitoneal (i.p.) single dose injection of 10, 56, or 100 mg/kg of glyphosate or AMPA, while one group (considered as the control) received an i.p. single dose of injectable water (220–240 μL). These doses of glyphosate and AMPA (i) were selected on the basis of a previous study from our group, where an acute dose of 100 mg/kg of AMPA induced a decrease in the activity of brain and skeletal muscle AChE [18]; (ii) do not represent a known dose observed during clinical exposure, nor are they based on a precise assessment of environmental risk (unknown in the case of AMPA); and (iii) allowed us to analyse some toxicodynamic aspects of these xenobiotics.

At post-injection day 5, rats were sedated with pentobarbital (≥100 mg/kg, i.p.), and a sample of blood was taken immediately by intracardiac punction. The blood was deposited on a heparinized tube and the plasma was obtained by a standard protocol of centrifugation. The measurements of cholinesterase activity were performed as soon as the plasma was obtained.

On the other hand, ten male bullfrogs (*Lithobates catesbeianus*) weighing 100–150 g were obtained from the vivarium of the Autonomous University of Aguascalientes. The frogs were pre-cooled by using ice water and then placed for 2 min into a freezer (as recommended elsewhere [19]), before sacrificing by decapitation on the day of the muscular contraction protocol (see Section 2.4) to dissect and obtain the rectus abdominis muscle.

All the experimental protocols in this study were approved by the Institutional Ethics Committee for the Use of Animals in Teaching and Research at the Autonomous University of Aguascalientes (CEADIUAA) and followed the Mexican Guidelines for Animal Care (NOM-062-ZOO-1999) and the National Research Council Guide for the Care and Use of Laboratory Animals [20].

### 2.3. Plasma Cholinesterase (ChE) Activity Method

The ChE activity was measured based on the Ellman method employing the Activity Assay Kit (MAK119, Sigma-Aldrich^®^) according to the instructions of the manufacturers. Briefly, 5 μL of plasma was used for colorimetric microassays, and the activity of plasma cholinesterase was calculated based on the product formed (thiocholine), originated by ChE activity, which reacts with 5,5′-dithiobis (2-nitrobenzoic acid). The reaction was made at pH 7.5 at room temperature, taking measures at min 0 and min 10. The absorbance measured at 412 nm in a Multiskan FC Microplate Reader (Thermo Scientific^®^, Shanghai, China) was proportional to the enzymatic activity of plasma cholinesterase and was calculated according to the following formula:

$$Enzimaticactivity\left(\mu molthiocholine/min\right)=\left(\frac{\left(Abs412\right)final-\left(Abs412\right)initial}{\left(Abs412\right)calibrador-\left(Abs412\right)blank}\right)\times N\times 200$$

where *N* is the dilution factor, and 200 is the equivalent activity of the kit’s calibrator.

### 2.4. Muscular Contraction Protocol

**Figure 1:**
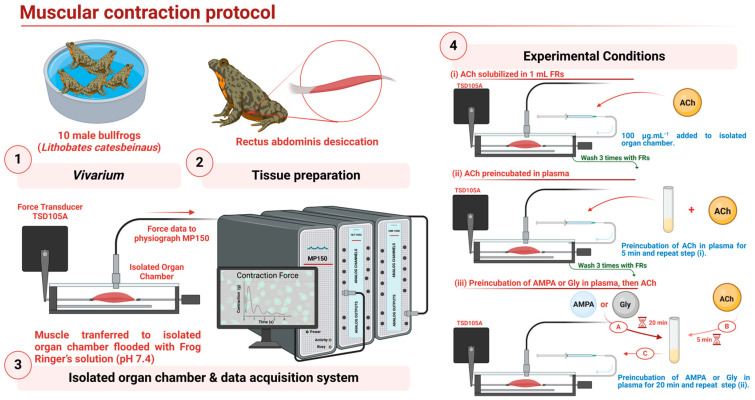
Muscular contraction protocol designed to study the effect of AMPA and glyphosate on human plasma cholinesterase activity. ACh, acetylcholine; AMPA, aminomethylphosphonic acid; FRs, Frog Ringer’s solution; Gly, glyphosate.

Muscular contraction measurements were carried out employing the rectus abdominis muscle from male bullfrogs. Briefly, 10 muscles desiccated from 10 decapitated frogs were placed individually into an isolated organ chamber flooded with Frog Ringer’s solution (FRs) pH 7.4. One litre of FRs consisted of [NaCl (6.5 g), KCl (0.14 g), NaH_2_PO_4_ × 2H_2_O (0.0065 g), glucose (2 g), NaHCO_3_ (0.4 g), CaCl_2_ (0.12 g)]. Initially, muscular contractions were recorded after addition of acetylcholine (ACh solubilized in 1 mL of FRs) to the chamber; next, to analyse the anticholinesterase action of plasma cholinesterase, 1 mL of human plasma was pre-incubated for 5 min with ACh and was added to the chamber; then, to study the effect of AMPA and glyphosate on muscular contraction, the following protocol was performed: AMPA or glyphosate solubilized in 1 mL of human plasma were incubated by 20 min. Immediately, ACh was added to the mix with another 5 min of incubation; this blend was finally added to the chamber (Figure 1).

For the previous preparations, the amount of ACh was 100 μg; AMPA and glyphosate 10 mg were used. Between each experimental condition, the muscle was washed 3 times with FRs to recover the baseline. The force measurements were amplified using the acquisition system WSW MP150 coupled to variable force transducer TSD105A at a sampling rate of 1000 Hz and employing the software Acqknowledge 4.1 MP150 (all from Biopac System Inc., Goleta, CA, USA). The plasma samples were obtained from 5 healthy donors (3 males and 2 females). The research protocol was approved by the ethical committee of the Autonomous University of Aguascalientes and was carried out in accordance with the code of ethics of the World Medical Association.

### 2.5. Statistical Analysis and Graphs

Statistical analysis and graphs were performed using the software GraphPad Prism version 9.02. The results are presented as mean ± S.D. Experimental data were analyzed using one-way ANOVA and Dunnett’s post hoc comparison or repeated-measures ANOVA. A value of *p* < 0.05 was considered statistically significant. Figure 1 was made with https://www.biorender.com/ with a license to BAM-C.

## 3. Results

**Figure 2:**
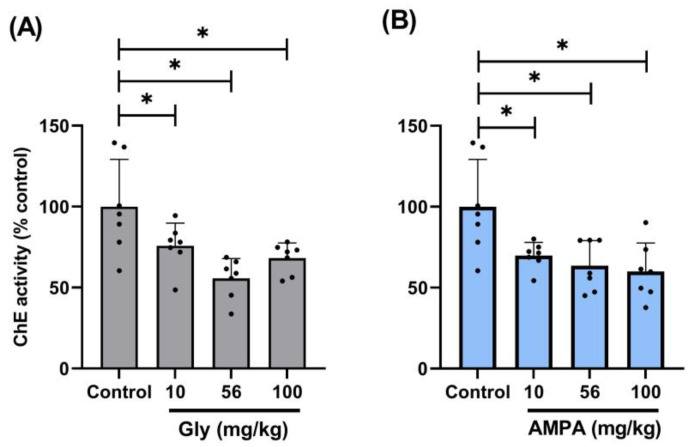
Plasma cholinesterase (ChE) activities obtained after acute intoxication with glyphosate (Gly; grey bars) or AMPA (blue bars)in rats. Single i.p. doses of AMPA (**A**) or Gly (**B**) at 10, 56, or 100 mg/kg decreased the plasma cholinesterase activity. Data are expressed as individual (black) dots and mean ± S.D. *n* = 7 per group. Note that “Control” data are the same in both (**A**) and (**B**). *, *p* < 0.05.

The acute exposure produced by single increasing doses of either AMPA or glyphosate (given i.p.) reduced (*p* < 0.05), to a similar extent, the rat plasma ChE activity compared to the control group that received an i.p. single dose of injectable water (220–240 μL; see Table 1 and Figure 2). No significant differences (*p* > 0.05) were observed between the ChE activity decrease produced by 10, 56, and 100 mg/kg of either AMPA or glyphosate.

**Figure 3:**
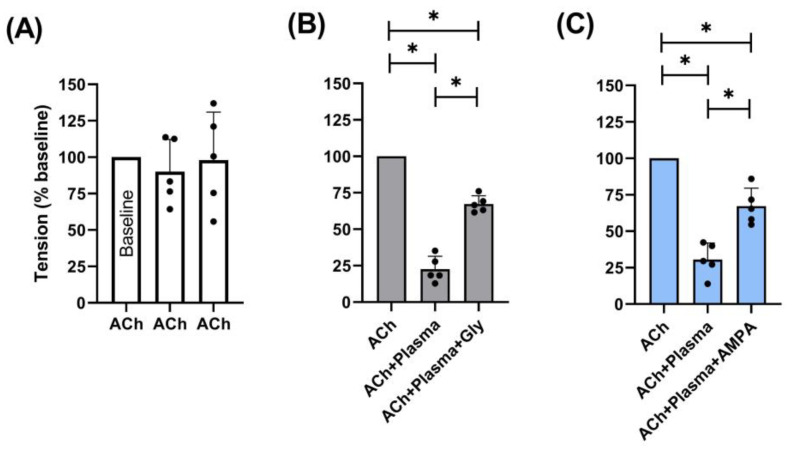
Effects of glyphosate (Gly; grey bars) or AMPA (blue bars) on muscular tension induced by acetylcholine (ACh) in frog’s rectus abdominis muscle. Graphs indicating the force contractions (tension) induced after consecutive stimuli with Ach pre-incubated with (**A**) nothing (control); (**B**) plasma or plasma + AMPA; and (**C**) plasma or plasma + Gly compared to the corresponding tension induced by ACh. Data are expressed as individual (black) dots and mean ± S.D. *n* = 5 per group. *, *p* < 0.05.

The above findings led us to explore the effects of both AMPA and glyphosate on human plasma ChE activity. In view that we cannot perform these experiments directly in humans, we decided to design an experimental protocol to analyse and compare the effects of AMPA and glyphosate on human plasma ChE by measuring the muscular contraction induced by ACh in the rectus abdominis muscle of frogs (see Section 2). This frog muscle was stimulated with ACh to activate the muscle-type nicotinic receptor located in the neuromuscular junction [21] to produce a measurable contraction (Figure 3).

Initially, the muscles were stimulated three times with ACh to evaluate the resulting contractions and potential tachyphylaxis; as shown in Figure 3A, these responses were highly reproducible as no significant changes were observed (*p* > 0.05). Then, the muscles were exposed to ACh dissolved in plasma to observe the effects of human plasma ChE on the ACh-induced tension, resulting in 25% of the one induced during baseline (indicating indirectly that plasma ChE degraded ACh).

Subsequently, the muscles were stimulated with the same ACh solution, but plasma was pre-incubated with AMPA or glyphosate, which resulted in a partial recovery of ACh-induced muscular tension; this finding suggests that both compounds decreased plasma ChE activity.

In the tension induced by ACh after exposure to plasma pre-incubated with AMPA (Figure 3B) or glyphosate (Figure 3C), ACh was 67.16 ± 11.07% and 67.28 ± 5.10% less intense, respectively, when compared to baseline. This finding implies that AMPA and glyphosate exerted a similar decrease in the plasma ChE activity.

## 4. Discussion

It has recently been reported that GBH exerts metabolomic changes in rat serum for four important metabolites (i.e., paraxanthine, epinephrine, L-(+)-arginine, and D-arginine); these metabolites could be involved in neurological changes as proposed by an ingenuity pathway analysis [22]. It remains to be determined if some of those metabolomic alterations may explain the discrete alterations in cognition and behaviour induced by GBH exposition [7]. Also, some in vitro assays suggest that glyphosate is a weak inhibitor of the activity of cholinesterase, with inhibition ranges between 11.0 and 17.6% [8,23,24]. Nevertheless, in vivo assays under subchronic exposure to GBH demonstrated a decreased AChE activity (about 50%) in a wide range of brain samples obtained from rats perinatally exposed to 100 and 200 mg/kg of GBH [10] or from mice subchronically and chronically exposed to 250 mg/kg of GBH [9].

It is noteworthy that the anticholinesterase action of AMPA has been little explored. With this in mind, as a first approach, we decided to employ the bullfrog skeletal muscle protocol (Figure 1) rather than a direct enzymatic assay in human plasma. This protocol allowed us to explore ex vivo the actions of AMPA and glyphosate after a brief incubation period in a functional phenomenon (i.e., the isolated organ) rather than a biochemical assay.

In vitro assays have reported that AMPA could act as a weak inhibitor of erythrocyte ChE since incubation of erythrocytes with 5 mM AMPA led to a decrease of 16.1% in this enzymatic activity [8]. Certainly, we do not know if i.p. doses of 100 mg/kg AMPA in our study reached plasma concentrations of 5 mM or higher. Nevertheless, since AMPA degradation is not well documented, we cannot categorically discard that AMPA metabolism in rats may generate other metabolites with a certain degree of anticholinesterase action.

Moreover, the half-life of plasma ChE is higher than 10 days in humans [25], while a wide spectrum (3 h to 15 days) has been reported in rats [26]; thus, the decrease in ChE activity after 5 days of exposure to AMPA/glyphosate (Table 1 and Figure 2) may suggest an irreversible direct (specific) rather than an indirect (unspecific) interaction of both compounds with ChE. Clearly, additional in vitro assays are required to further confirm this view.

To our knowledge, no study has reported the in vivo effects of AMPA on plasma ChE. On this basis, our experiments show that incubation of glyphosate and its metabolite AMPA in human plasma decreased the activity of ChE (Figure 3). In agreement with this finding, some in vitro studies have reported that AMPA and glyphosate behave as weak inhibitors of plasma ChE [8]. Indeed, since glyphosate belongs to the organophosphorus (OP) family, the cholinesterase activity has been used as a biomarker of OP pesticide exposure [27,28].

The global agricultural use of glyphosate has increased in the last decades [29]. Even its use in other activities, such as gardening in residential areas, could contribute to possible urban exposure to glyphosate and AMPA [30]. In this sense, the negative effects of glyphosate on human health have been widely reported [7]; nevertheless, the potential toxicity of its metabolites must be considered.

In relation to the above facts, AMPA, the main metabolite of glyphosate, (i) has a half-life three times longer than that of glyphosate in soils [11]; and (ii) it is also produced as a degradative metabolite of other common daily compounds, such as polyphosphonate-detergents [17]. This predisposes to a possible environmental risk that must be addressed. Indeed, both glyphosate and AMPA were commonly found in urban streams and wetlands in Australia [31], raising concerns also in the United States [32,33], the European Union [34,35], and Asia [36].

Considering the above lines of evidence, in the present study, we have further demonstrated that acute exposure to AMPA can induce a decrease in ChE activity in both human and rat plasma. Obviously, additional experiments would have to be carried out to better characterize the toxicity of AMPA (i.e., a comparative analysis of subchronic exposure vs. chronic exposure).

### Study Limitations

Admittedly, apart from its scope, our study may have some limitations. For example:

(i) The i.p. doses employed to evaluate the effects of AMPA and glyphosate on human plasma ChE (Table 1) were higher than those used in humans after oral ingestion [37]. Hence, the influence of the difference in pharmacokinetic factors in these studies remains unknown. The role of pharmacokinetics may also help explain why no clear dose-dependence was observed with the effects of these compounds (Table 1 and Figure 2).
(ii) Our in vitro results (Figure 2) “fall beyond the role of pharmacokinetic factors in view that, unlike in vivo studies, the in vitro models generally allow: (a) the exclusion of nervous and hormonal influences; and (b) the control of most experimental factors (including concentrations, temperature, maximum responses, etc.) to guarantee that equilibrium conditions are reached”, as explained in detail elsewhere [38].
(iii) The lack of a time-course analysis and a whole concentration–response curve of AMPA/glyphosate in the protocol of decreased human ChE activity (Figure 3); both analyses would allow us to know more details about the type of molecular interactions exerted by these xenobiotics.
(iv) The AChE activity assay Kit (MAK119; purchased from Sigma-Aldrich, as indicated below in Section 2.1) indicates that its substrates may also be susceptible to the action of acetylcholinesterase; thus, strictly speaking, our results showing ChE activity include the total activity of at least these two enzymes. This is why our results report ChE (not butyrylcholinesterase) activity.

## 5. Conclusions

Our results indicate that AMPA, like glyphosate, decreased ChE activity in the plasma of rats (when given i.p.) and humans (when added in vitro). This view opens the possibility that both compounds display comparable toxicodynamics and may have similar toxicological effects, highlighting the importance of in-depth investigations with AMPA.

## Figures and Tables

**Table 1:** Plasma cholinesterase (ChE) activity (μmol/min per L) in plasma of rats acutely exposed to AMPA or glyphosate given i.p. Note that the control data (0 mg/kg) in both AMPA and glyphosate are the same. *, *p* < 0.05 vs. control.

Drug	Doses (mg/kg; i.p.)			
	0 (Control)	10	56	100
AMPA	939 ± 253	656 ± 70 *	597 ± 134 *	563 ± 153 *
Glyphosate	939 ± 253	640 ± 122 *	522 ± 107 *	712 ± 81 *

## Data Availability

Data will be made available on request by correspondence to: bruno.marichal@edu.uaa.mx.
